# Breast cancer patients with a pre-existing mental illness are less likely to receive guideline-recommended cancer treatment: A systematic review and meta-analysis

**DOI:** 10.1016/j.breast.2024.103855

**Published:** 2024-12-07

**Authors:** Katie Elliott, Emily Haworth, Iakov Bolnykh, R. Hamish McAllister-Williams, Alastair Greystoke, Adam Todd, Linda Sharp

**Affiliations:** a Population Health Sciences Institute, Newcastle University, Newcastle University Centre for Cancer, Newcastle upon Tyne, England, UK; b Translational and Clinical Research Institute, Newcastle University, Newcastle upon Tyne, UK; c Cumbria, Northumberland, Tyne and Wear NHS Foundation Trust, Newcastle upon Tyne, UK; d Newcastle Biomedical Research Centre, Newcastle University, Newcastle upon Tyne, UK; e School of Pharmacy, Newcastle University, Newcastle Upon Tyne, UK; f Newcastle Upon Tyne Hospitals NHS Foundation Trust, Newcastle Upon Tyne, UK

**Keywords:** Breast cancer, Treatment, Mental illness, Health disparities, Comorbid conditions, Oncology, Psychiatry

## Abstract

Breast cancer is the most commonly diagnosed cancer worldwide, with early detection and advanced treatments contributing to declining mortality rates. However, managing comorbid conditions, particularly mental illness, presents significant challenges for cancer treatment. This study systematically reviews and meta-analyses the impact of having a pre-existing mental illness on breast cancer treatment utilisation, focusing on specific treatments and comparing different mental illnesses. MEDLINE, EMBASE, CINAHL, and APA PsycInfo databases were searched. After screening, fifteen studies were identified as meeting the inclusion criteria. The included studies were predominantly from high-income countries, and compared breast cancer treatment in patients with and without pre-existing mental illnesses including anxiety, mood disorders, schizophrenia and psychotic disorders, and neurodevelopmental disorders. Meta-analysis revealed that patients with mental illnesses were significantly less likely to receive guideline-recommended treatments (OR = 0.78, 95 % CI 0.72–0.83, N = 5), chemotherapy (OR = 0.56, 95 % CI 0.34–0.78, N = 6), or radiotherapy (OR = 0.79, 95 % CI 0.66–0.93, N = 5). They were also significantly more likely to undergo mastectomy instead of breast-conserving surgery (OR = 1.38, 95 % CI 1.24–1.52, N = 4). Findings were consistent across different mental illnesses. This review highlights the need for targeted interventions to improve healthcare access and address provider biases, promoting better integration of mental health and oncology care.

## Highlights

• Patients with a mental illness have reduced receipt of guideline-recommended breast cancer treatment, chemo and radiotherapy.
• Patients with mental illnesses are more likely to have a mastectomy over breast-conserving surgery.
• Disparities in treatment receipt persist across anxiety disorders, mood disorders and psychotic disorders.
• The study highlights gaps in care, urging strategies to ensure equitable treatment for patients with mental illnesses.
• Integrating mental health and oncology care is key to improving outcomes and reducing mortality in high-risk groups.

## 1 Background

Cancer has a major impact on public health and is a leading cause of premature mortality. Cancer incidence is increasing, and the burden is predicted to rise further with population ageing [1]. Breast cancer is the most common cancer worldwide, with over 2.3 million new cases and 685,000 deaths in 2020 [2], and a lifetime incidence of around 1 in 7 women in the UK [3]. In contrast to some other cancer types, when breast cancer is detected early it is highly treatable and, in some cases, is completely curable [4]. The introduction of mass-scale mammography-based screening programmes has promoted earlier breast cancer detection and this, alongside advances in pharmacological treatments, means breast cancer mortality rates are steadily declining [5,6].

Holistically and successfully managing patients who in addition to cancer suffer from other long-term conditions can be a major challenge; it has been consistently shown that cancer patients with additional long-term conditions have significantly poorer survival rates [7]. These conditions may both directly and indirectly affect survival through their impact on underlying biology and patient health, and because the conditions (and their management) may influence cancer treatment decisions. There is potential for drug interactions with cancer treatment(s), as well as concerns about tolerability, compliance, and ability to safely complete planned cancer treatment(s). This complexity may prevent access to optimal cancer treatment.

A mental illness may complicate cancer treatment for many, as up to 25 % of the population are affected by one or other such disorders every year [8]. Some mental illnesses occur considerably more frequently in women, including the most common - depression and anxiety disorders [9]. Although cancer incidence rates are similar in people with and without mental illness, it has been consistently reported that people with a mental illness have poor cancer outcomes; specifically they are significantly more likely to die from cancer and/or survive for a shorter period following diagnosis [10]. Breast cancer mortality risk is consistently reported to be significantly higher in patients with a mental illness, even after controlling for confounders such as age, year of diagnosis and comorbidities [11,12].

**Fig. 1:**
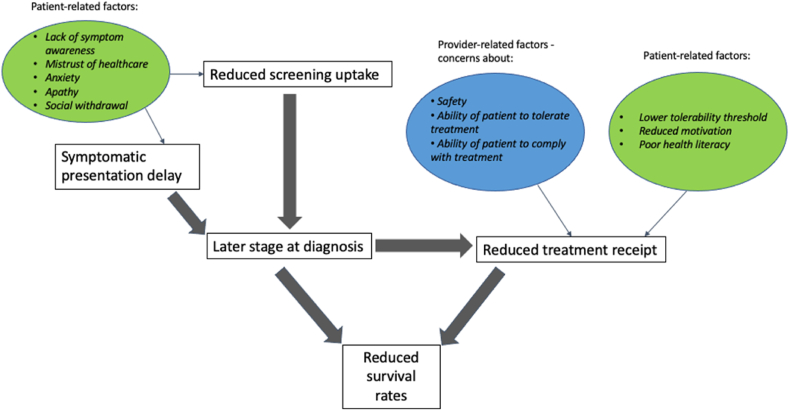
Pathway diagram showing the interaction between the key factors contributing to reduced survival rates in breast-cancer patients with a pre-existing mental illness.

Despite the evidence of a significant mortality gap existing in breast cancer patients with a pre-existing mental illness, the specific factors responsible for this, their relative contribution, and whether these factors vary between different mental illnesses is not yet fully understood, although several contributing factors have been suggested. Firstly, there is evidence suggesting that disparities exist in breast cancer screening – with a recent meta-analysis evidencing an overall 29 % reduced odds of receiving a mammogram in those with a mental illness [13]. Subgroup analysis showed that this reduction in screening uptake was even greater in those with severe mental illnesses, such as psychotic disorders (50 %). Secondly, it has been reported that people with a pre-existing mental illness are more likely to be diagnosed with breast cancer at a more advanced stage [14]. This is important, given more advanced or metastatic cancer is associated with worse survival. However, previous studies exploring these disparities have found that stage at diagnosis only explains a small proportion of the excess mortality observed in breast cancer patients with a pre-existing mental illness [11,12,15,16]. This therefore indicates that factors operating post-diagnosis may play an important role. A third possible explanation for this mortality gap, therefore, is the existence of disparities in breast cancer treatment utilisation. There is some research to suggest that breast cancer patients with a pre-existing mental illness are less likely to receive guideline-recommended treatment and are at higher risk of not completing allocated treatment courses [17]. Moreover, individuals with a mental illness may also have an increased risk of experiencing delays to accessing treatment [17]. Fig. 1 summarises the relationship between key factors contributing to the worse breast cancer outcomes in patients with a mental illness.

The primary aim of this systematic review is to assess whether there are significant differences in the receipt of each of the different types of breast cancer treatment in women with and without a pre-existing mental illness. The secondary aim is to assess whether this association/strength of association varies between different types of mental illness. The ultimate goal is to establish where disparities occur to inform areas to target for future intervention.

## 2 Methods

### 2.1 Search strategy and study selection

To address the research question, the systematic review sought to identify studies which investigated breast cancer treatments utilisation in patients with and without a pre-existing mental illness. The review was registered with PROSPERO (CRD42023413962) and reported following the PRISMA guidance. This review focused on four groups of mental illness: anxiety and depressive disorders; schizophrenia and psychotic disorders; bipolar disorder; and neurodevelopmental disorders. For studies to be eligible for inclusion in the review, the mental illnesses could have been either self-reported or ascertained from medical records prior to receiving a cancer diagnosis. Any other mental illnesses, and those which had been diagnosed after receiving a cancer diagnosis, were excluded. For studies which included data on people with eligible and other (ineligible) mental illnesses, only data on the mental illnesses of interest was extracted. The inclusion criteria were defined by the PICOS framework (Table 1).

**Table 1:** Summary of PICOS eligibility criteria.

Population	Women with a diagnosis of invasive or *in-situ* breast cancer, with and without a (pre-cancer) diagnosis of the following mental health conditions: anxiety and/or depression; schizophrenia and other psychotic disorders; neurodevelopmental disorders; and bipolar disorder. Only women were eligible for inclusion given breast cancer is rare in men.
Intervention	Any intervention initiated for the treatment of breast cancer including but not limited to cancer-directed surgery (any and type), chemotherapy, radiotherapy, endocrine therapy, biological and precision therapy or immunotherapy, receipt of ‘guideline-appropriate’ treatment. Data on other treatment delivery variables such as treatment delays and adherence were also included were appropriate.
Comparator	Interventions compared between participants with and without a pre-existing mental illness.
Outcome	Breast cancer treatment according to pre-existing mental illness status.
Study design	Observational quantitative studies including but not limited to: prospective cohort, retrospective cohort, cross-sectional and case-control studies.

A bibliographic database search was conducted using Ovid MEDLINE and In-Process, In-Data-Review & Other Non-Indexed Citations (1946 to April 2024), Embase (1974 to April 2024) and CINAHL (1961 to April 2024). Searches were conducted using combinations of MeSH terms and text words for mental illnesses of interest, breast cancer and cancer treatment(s). Full search strategies are presented in Supplementary file 1. Articles and abstracts published in English between 1^st^ Jan 1995 and 10^th^ April 2022 were eligible for inclusion.

Studies were excluded if they: [1]: were not published in English [2]; were intervention studies such as randomized controlled trials [3]; did not include a comparison group (i.e. women who had breast cancer and no mental illness pre-cancer diagnosis) [4]; were systematic reviews and/or meta-analyses [5]; were looking only at another part of the cancer pathway (e.g. screening, diagnosis or survival).

Double independent screening of titles and abstracts was completed by two members of the research team (KE and EH or IB) to identify those which were potentially eligible. Any disagreements after this stage were discussed within other authors (AT, LS) to reach consensus. Full text screening was then completed independently by three authors (KE, EH and IB). Any uncertainties or disagreements were discussed with the rest of the review team to reach consensus. Forwards and backwards citation searching were also undertaken on included studies to identify additional relevant articles.

### 2.2 Data extraction and quality assessment

Data was extracted by one author (KE) and checked in full by another (EH). In instances of missing or inconsistent data, study authors were contacted by email. If authors did not respond after one month, extraction was based on the data reported in the paper. Data were extracted on: author(s), publication year, country, study design, data source, number in study population, mental illness type(s), age at diagnosis, socio-economic status, ethnicity/race, cancer stage at presentation/diagnosis, type of cancer treatment received, time to treatment, comorbidities (e.g. Charlson comorbidity score), and relevant statistics including *p* values, odds ratios and hazard ratios where available. The data source, time period and age group of participants in each study were compared following data extraction to identify any overlap of data between included studies. Eligibility criteria for studies to be included in the meta-analysis were as follows: (i) data for a mental illness vs no mental illness for one treatment outcome (yes/no); and (ii) an independent sampling frame/data source. Where multiple papers used data from the same sampling frame/data source with an overlap in time period, the publication with the largest sample size was entered into the meta-analysis.

A modified version of the ISPOR checklist for retrospective database studies was used to assess the quality of included studies [18]. Particular attention was paid to data sources, statistical results of interest, and the generalisability/applications of the findings [19]. This was completed by one author (KE) and checked in full by another (EH).

### 2.3 Data/evidence synthesis

Data was synthesised using a summary of findings table. Percentages of patients receiving different cancer treatments by mental illness status were calculated from raw data in instances where this was not reported. Unadjusted OR for treatment receipt in those with vs without a mental illness were computed by the review authors if not reported; this maintained consistency across studies as some studies performed adjusted analyses but tended to adjust for different variables.

Meta-analyses were performed on eligible studies with random-effects models used the Mantel-Haenszel approach and the generic inverse variance method, to computed pooled odds ratios for likelihood of treatment receipt by mental illness status. Heterogeneity assessment was performed using the I^2^ and Tau-squared statistics [20]. Studies were combined for meta-analysis when at least two reported on the same outcome, and subgroup meta-analysis was performed where mental illnesses between studies could be consistently categorised. Meta-analysis was conducted in Stata 18.0.

## 3 Results

### 3.1 Search results

**Fig. 2:**
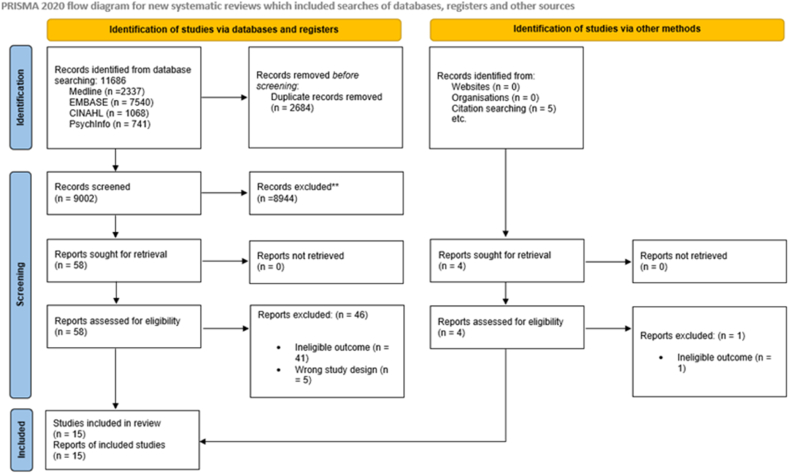
PRISMA 2020 flow diagram.

The search identified 11,686 citations and after removal of duplicates and title and abstract screening, 58 papers progressed to the full-text review stage (PRISMA flowchart in Fig. 2). After full text checking, 15 papers met the criteria for inclusion in the final synthesis.

### 3.2 Study characteristics

As shown in Table 1, just over half of the included studies were from the United States (US) (N = 8 studies) [[21], [22], [23], [24], [25], [26], [27], [28]], with the rest from Japan (N = 2) [29,30], Denmark (N = 2) [31,32] Finland (N = 1) [11] and France (N = 2) [33,34]. Across included studies data was compared on the following treatment-related variables: receipt of guideline-appropriate treatment (N = 5) [22,24,27,29,32] adjuvant radiotherapy (N = 5), chemotherapy (N = 6) [22,28,30,32,33], type of surgery (mastectomy vs breast-conserving surgery) (N = 4) [26,30,32,33], implant vs autologous reconstruction modality (N = 1) [21], endocrine therapy utilisation (N = 5) [25,28,30,32,33], and treatment delays (N = 1) [23]. 13/15 studies used population-based cancer registry data. For mental illnesses, there was heterogeneity in the conditions included: some studies focused on just one mental illness, while others included multiple types. In relation to the categories of mental illness of interest, 11 studies included data on patients with schizophrenia/psychosis [11,21,[23], [24], [25],[28], [29], [30],[32], [33], [34]], 10 on depression and/or anxiety [11,[21], [22], [23], [24], [25], [26], [27], [28],^31^,^34^], and seven on bipolar disorder [11,21,[23], [24], [25],^33^,^34^] (Table 2). Fourteen studies reported mental illness based on the presence of a psychiatric diagnosis in the patients’ medical records, whereas one study used self-reported data from the Medicare Health Outcomes Survey (MHOS) to determine the presence of depressive symptoms [26].

**Table 2:** Characteristics of included studies and summary of findings on the associations between mental illness and different breast cancer treatment receipt variables.

Study characteristics					Outcomes and findings			
Authors, year (country)	Data source(total sample size)	Breast cancer diagnosis and date)	Mental disorder(s) of relevance	Mental disorder assessment measure	Relevant treatment elements assessed	Unadjusted OR/RR (95 % CI) ^[^a^]^	Adjusted OR/RR (95 % CI) (MI vs no MI)	Adjustment variables
Ahlgrén-Rimpiläinen et al., 2020 [10] (Finland)	Finnish cancer registry (n = 80,671)	Women with breast cancer diagnosis (ICD-O-3 code C50) in 1990–2013	Non-affective psychosis Mood disorders	Hospital Discharge Register (HDR) ICD-10 codes between 1969 and 2013/Hospital admissions due to SMI at least a year before cancer diagnosis or earlier.	Receipt of radiotherapy vs not	OR = 0.73 (0.68–0.79)	N/A	N/A
Buscariollo et al., 2019 [26] (USA)	SEER-MHOS database (n = 1407)	Women ≥65 years old with DCIS or stage I BC diagnosed 1998–2011	Pre-diagnosis depressive symptoms according	Measured using an algorithm of Medicare Health Outcomes Survery (MHOS) responses derived from the Diagnostic Interview Schedule	Associations with treatment choice (BCS and radiation therapy, BCS alone, mastectomy)	Combined DCIS and stage 1 BC: Mastectomy: OR = 1.20 (0.90–1.61) Radiotherapy: OR = 0.75 (0.58–0.97)	Mastectomy: DCIS - OR = 1.88 (0.91–3.86) Stage 1 BC – OR = 1.03 (0.69–1.53) Radiotherapy: DCIS – OR = 0.49 (0.25, 0.96) Stage 1 BC – OR = 0.78 (0.49, 1.22)	Age at diagnosis, race and ethnicity, smoking status, marital status, education, income, comorbidities, geographic region, year of diagnosis, hormone receptor status, tumor grade, tumor size
Dalton et al., 2018 [32] ^[^b^]^ (Denmark)	Denmark central cancer registry (n = 56,152)	Women who underwent surgery for early stage breast cancer between 1995-2011	Schizophrenia or related disorders	Hospital contacts including ICD8/10 codes for prior to breast cancer diagnosis	Allocated to guideline treatment, surgery received (mastectomy lumpectomy, biopsy), adjuvant therapy initiated (endocrine therapy, chemotherapy, endocrine and chemotherapy, none).	Guideline-recommended: OR = 0.95 (0.75–1.20) Mastectomy: OR = 1.47 (1.23–1.76) Chemotherapy: OR = 0.27 (0.20–0.37) Endocrine therapy: OR = 0.99 (0.83–1.18)	N/A	N/A
Fond et al., 2021 [34] (France)	French national hospital database (n = 38,612)	Women who died of breast cancer in French hospitals (2014–2018)	Bipolar disorder, recurrent major depression, schizophrenia	ICD-10 diagnositic codes in acute and/or psychiatric datatbases in the 4 years before death	Chemotherapy in the last 14 days of life	OR = 0.70 (0.60–0.81)	OR = 0.70 (0.60–0.83)	age at death, social deprivation, year of death, survival time, metastases, Charlson modified comorbidity index, smoking addiction and hospital category.
Goodwin et al., 2004 [22] (USA)	Medicare SEER database (n = 24,696	Women diagnosed with incident breast cancer (1993–1996)	Depression	ICD-9 diagnosis in the 2 years prior to breast cancer diagnosis	Receipt of guideline-appropriate treatment Receipt of chemotherapy vs not	OR = 0.76 (0.69–0.83) OR = 0.66 (0.55–0.80)	OR = 0.84 (0.75–0.94)	Age, ethnicity, comorbidity
Haskins et al., 2019 [25] (USA)	SEER medicare database (n = 21,894	Women aged 68+ surgically treated for stage I-IV ER + breast cancer (2007–2013)	Unipolar depression Anxiety Bipolar depression Schizophrenia Non-schizophrenia psychoses	Mental illness diagnosed within 36 months prior to breast cancer diagnosis:	Endocrine therapy adherence, initiation and discontinuation	Initiation: Significantly more common in patients with unipolar depression HR = 0.95 (0.90–0.99), Bipolar depression HR = 0.85 (0.74–0.98), and Non-schizophrenia psychosis – HR = 0.89 (0.83–0.96) Discontinuation: (stopping endocrine therapy before 5 years) significantly more likely in anxiety - HR = 1.24 (1.13–1.37) Bipolar depression – HR = 0.95 (0.71–1.28) Schizophrenia – HR = 0.81 (0.56–1.18) Non-schizophrenia psychosis - HR = 1.20 (1.04–1.37) Adherence: significant difference in proportion of days covered in patients with schizophrenia – estimate = 0.035 (0.007, 0.062)	Initiation: significantly less common in patients with unipolar depression HR = 0.95, (0.90–0.99), bipolar depression HR = 0.85, (0.74–0.98) and non-schizophrenia psychosis (HR 0.89, 0.83–0.96). Discontinuation: significantly more common in patients with anxiety HR = 1.24 (1.13–1.37), non-schizophrenia psychotic (HR = 1.20, 1.04–1.37) disorders.	stage, age, year of diagnosis, race, ethnicity, and 3-year NCI modified comorbidity index
Iglay et al., 2017 [23] (USA)	SEER medicare database (n = 16,636)	Women diagnosed with stage II – IIIa breast cancer (2005–2007)	Anxiety Depression Anxiety and depression Severe mental illness (Bipolar disorder, Schizophrenia or other psychotic disorder).	ICD-9 code recorded for inpatient or outpatient claims during 3 years prior to breast cancer diagnosis	Initial treatment delay Adjuvant chemotherapy delay Adjuvant radiotherapy delay Surgery	N/A	Initial treatment delay >60 days from symptom recognition Any mental illness – 0.98 (0.87–1.10) Severe mental illness - RR = 1.36 (1.06–1.74) Anxiety only – RR = 0.92 (0.76–1.12) Depression only – RR = 0.96 (0.79–1.15) Anxiety and depression – RR = 0.89 (0.70–1.13) >90 days from symptoms recognition Any mental illness – RR = 1.08 (0.90–1.29) Severe mental illness – RR = 1.39 (0.95–2.04) Depression only – RR = . 1.08 - (0.82–1.42) Anxiety and depression – RR = 1.07 (0.76–1.52)	Age, income, comorbidity, race, ethnicity, SEER stage, marital status, AFCC stage
Kaneshiro et al., 2022 [29] (Japan)	St Mary's Hospital patient database (n = 665)	January 2010 – Feburary 2020	Schizophrenia	diagnosis by a psychiatrist according to ICD-10	Recommended cancer treatment execution rate	OR = 0.43 (0.24–0.76)	N/A	N/A
Lawrence et al., 2021 [28] (USA)	New York State Cancer Registry (n = 8670)	Women aged <65 years diagnosed with breast cancer (2004–2016)	Schizophrenia Depression	At least three relevant claims for mental illnesses with at least one claim within three years before breast cancer diagnosis, as determined by ICD-9 classification	Receipt of: chemotherapy, radiotherapy, endocrine therapy, surgery	Chemotherapy: OR = 0.84 (0.75–0.89) Radiotherapy: OR = 0.75 (0.70–0.80) Endocrine therapy: OR = 1.22 (1.12–1.32) Surgery: OR = 1.15 (0.99–1.32)	N/A	N/A
Lei et al., 2022 [27] (USA)	The Kentucky Cancer Register (n = 988)	Women aged 20 years + diagnosed with primary invasive breast cancer (2007–2011)	Depression	ICD-9 code recorded for inpatient or outpatient claims from 1 year prior (pre-diagnosis) to 1 year after cancer diagnosis (persistent)	Receipt of guideline-recommended breast cancer treatment	Pre-diagnosis and persistent depression (combined OR = 0.92 (0.76–1.12)	Pre-diagnosis depression: OR = 0.75 (0.54–1.04) Persistent depression: OR = 0.95 (0.69–1.32)	age at cancer diagnosis, smoking status, race (White, Black, or other), Appalachian status, marital status (married, never married, separated/divorced/widowed, or unknown), primary insurance payer at the time of diagnosis (Medicare, Medicaid, or private insurance), census‐tract level educational attainment, census‐tract level percent below poverty, and comorbidity status
Mahabaleshwarkar et al., 2015 [24] (USA)	Medicaid Analytic Extract files (n = 2142)	Women aged 18–65 diagnosed with breast cancer in 2007	Mood disorders Psychotic disorders	ICD-9-CM codes associated with medical records in 12 months prior to breast cancer diagnosis	Receipt of guideline-consistent breast cancer treatment	Mood disorders: OR = 0.76 (0.60–0.97) Psychotic disorders: OR = 0.97 (0.67–1.43)	Mood disorders: OR = 0.75 (0.59–0.97) Psychotic disorders: OR = 0.94 (0.65–1.39)	Age at diagnosis, race, the type of reimbursement system, breast cancer stage at diagnosis, Charlson comorbidity index, location of residence, state of residence, the number of outpatient visits in the last 12 months prior to the diagnosis of breast cancer.
Mehta et al., 2020 [21] (USA)	Patients who completed the breast-Q survey from a “large tertiary hospital” (n = 471)	Women who underwent breast reconstruction surgery (2013–2018)	Depression Bipolar disorder Schizophrenia Psychosis	A psychiatric diagnosis from medical records	Autologous vs implant-based breast reconstruction surgery	OR = 0.56 (0.35–0.89)	OR = 0.49 (0.28–0.84)	Presence of psychiatric diagnosis, age, BMI, ASA classification, preoperative radiation, preoperative chemotherapy, cancer stage
Seppanen et al., 2023 [33] (France)	système national données de santé (n = 97,760)	Incident treated breast cancer (2013–2014)	Psychotic disorders Bipolar affective disorders	Women ICD-10 codes for a a pre-existing severe mental illness 1 year prior to cancer diagnosis	Receipt of: chemotherapy, radiotherapy, endocrine therapy, surgery (mastectomy vs lumpectomy).	Radiotherapy: OR = 0.80 (0.69–0.92) Chemotherapy: OR = 0.78 (0.68–0.89) Endocrine therapy: OR = 0.88 (0.77–1.01) Mastectomy: OR = 1.38 (1.20–1.58)	Radiotherapy: OR = 0.87 (0.75–0.98) Chemotherapy: OR = 0.80 (0.70–0.91) Endocrine therapy: OR = 0.86 (0.75–0.99)	CMU-C/ACS status, FDep quintile at the place of residence, MRMI synthetic comorbidity index, and type of hospital where first breast cancer treatment was received
Shinden et al., 2017 [30] (Japan)	Kagoshima University Hospital Patient Database (n = 773)	Women with a primary breast cancer diagnosis/without distant metastasis who underwent curative surgical treatment (September 1992–January 2015)	Schizophrenia	A formal diagnosis	Mastectomy vs BCS Chemotherapy receipt Radiotherapy receipt Endocrine therapy receipt	OR = 2.55 (1.09–5.95) OR = 0.03 (0.00–0.50) OR = 0.16 (0.02–1.17) OR = 1.11 (0.51–2.42)	N/A	N/A
Suppli et al., 2020 [31] (Denmark)	Danish Psychiatric Central Research Register (n = 45325)	Women with a diagnosis of early stage breast cancer (1998–2011)	Depression	ICD-8 diagnosis 3 months prior −3 years prior to breast cancer diagnosis	Allocation to and initiation of guideline adjuvant systemic therapy	Guideline-appropriate treatment: OR = 0.69 (0.74–0.84) Chemotherapy: OR = 0.06 (0.06–0.06)	N/A	N/A

a ORs/HRs show likelihood of treatment receipt in patients with a mental illness compared to those without.

b Potential overlap in data sources for [31,32].

### 3.3 Quality appraisal and risk of bias

The included studies generally scored highly in terms of quality appraisal: scores ranged from 3.5 to 9 out of a possible 9, with a mean score of 8. Issues associated with data sources, study populations, and discussion of findings were generally well addressed. Lower scoring questions pertained to statistical analysis when determining associations between mental illness and treatment receipt (e.g., ORs) - 11 studies reported effect estimates, four did not. Similarly, the scoring related to undertaking an adjusted analysis was mixed; 9/15 studies performed an adjusted analysis).

### 3.4 Meta-analysis

The meta-analysis included data from twelve studies [11,22,[24], [25], [26], [27], [28], [29], [30],[32], [33], [34]]. Data from three of the studies were not included in the meta-analysis; one was excluded due to overlap of study population [31] with another included study [32]. The other two were single studies reporting on treatment delay [23] and comparing implant vs autologous breast reconstruction modality [21], therefore meta-analysis was not possible.

### 3.5 Receipt of ‘guideline-recommended’ breast cancer treatment

There were six studies that specifically compared the receipt of guideline-recommended breast cancer treatment in patients with, and without, pre-existing mental illness [22,24,27,29,31,32] Two of these studies compared treatment receipt in patients with depression to those without any history of depression [22,27], two studies focused on patients with schizophrenia [29,32], one study focused on depression and severe mental illness, while the other included data on patients with mood and psychotic disorders [21]. All six studies found that patients with a pre-existing mental illness were significantly less likely to receive guideline-recommended treatment. Due to a potential overlap of data source between the two Danish studies [31,31,32] was excluded from meta-analysis being the smaller of the two studies.

**Fig. 3:**
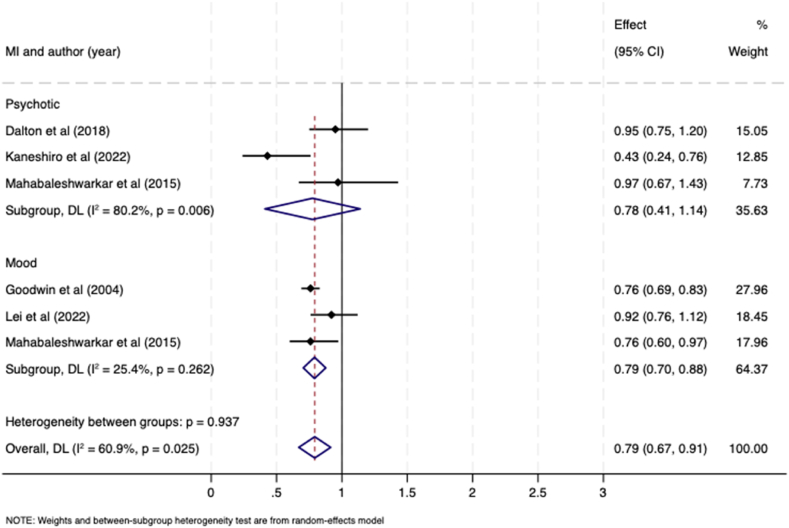
Meta-analysis forest plot showing odds ratio for likelihood of receiving guideline-recommended breast cancer treatment in women with a pre-existing mental illness compared to women without a mental illness, by type of mental illness.

The pooled overall OR for receipt of guideline-recommended treatment in those with a pre-existing mental illness compared to those without was 0.78 (95 % CI 0.72–0.83; I^*2*^ = 60.9 % (Fig. 3). Subgroup analysis by mental illness type showed the pooled OR for psychotic disorders was 0.77 (95 % CI = 0.61–0.92; I^*2*^ = 80.2 %; Tau^2^ = 0.08), and for mood disorders was 0.78 (95 % CI 0.72–0.84; I^*2*^ = 25.4 %; Tau^2^ = 0.00) with no evidence for heterogeneity between groups (p = 0.937).

### 3.6 Adjuvant chemotherapy

**Fig. 4:**
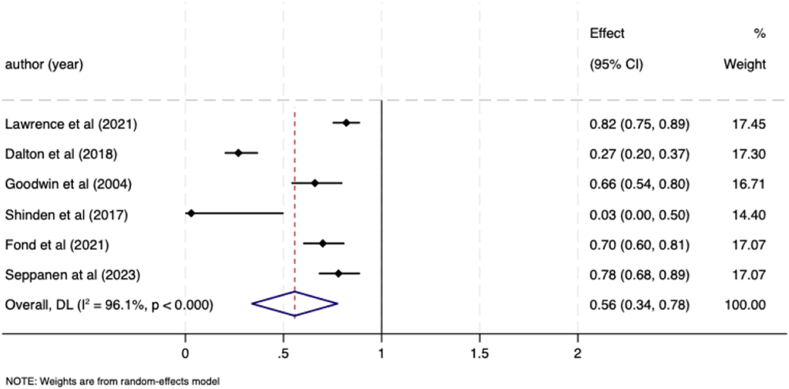
Meta-analysis forest plot showing odds ratio for likelihood of receiving chemotherapy in breast cancer patients with a pre-existing mental illness compared to women without a mental illness.

Six studies specifically compared receipt of adjuvant chemotherapy between breast cancer patients with, and without, a pre-existing mental illness and were all eligible for meta-analysis. These studies included data on patients with schizophrenia [26], depression [22], depression and severe mental illness [25], psychotic and bipolar affective disorders [33] and severe mental illnesses [29], schizophrenia and depression [28]. Those patients with a pre-existing mental illness were significantly less likely to receive adjuvant chemotherapy than those patients without a mental illness; the pooled OR was 0.56 (95 % CI 0.34–0.78; I ^*2*^ = 96.1 %; Tau^2^ = 0.07). (Fig. 4).

### 3.7 Adjuvant radiotherapy

**Fig. 5:**
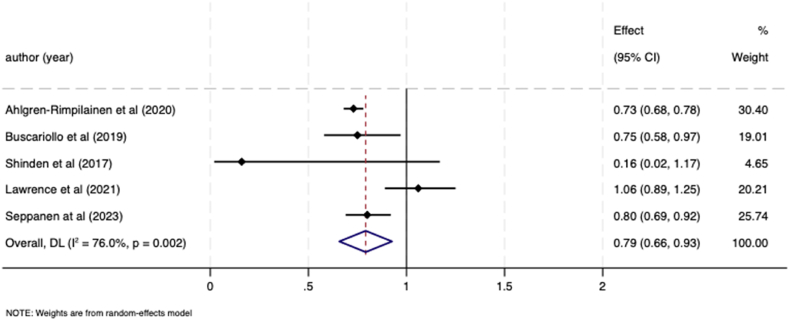
Meta-analysis forest plot showing odds ratio for likelihood of receiving radiotherapy in breast cancer patients with a pre-existing mental illness compared to patients without a mental illness.

Five studies specifically compared receipt of radiotherapy between breast cancer patients with and without a pre-existing mental illness and all were eligible for meta-analysis. These studies included patients with non-affective psychosis and mood disorders [11], schizophrenia and intellectual disability [30], history of depression [26], schizophrenia and depression [28] and severe mental illness [33]. Again, the analysis demonstrated that patients with pre-exiting mental illness were significantly less likely to receive treatment. The pooled OR for receipt of radiotherapy in those with a pre-existing mental illness compared to those without was 0.79 (95 % CI 0.66–0.93; I ^*2*^ = 76 %); Tau ^2^ = 0.02) (Fig. 5).

### 3.8 Adjuvant endocrine therapy

**Fig. 6:**
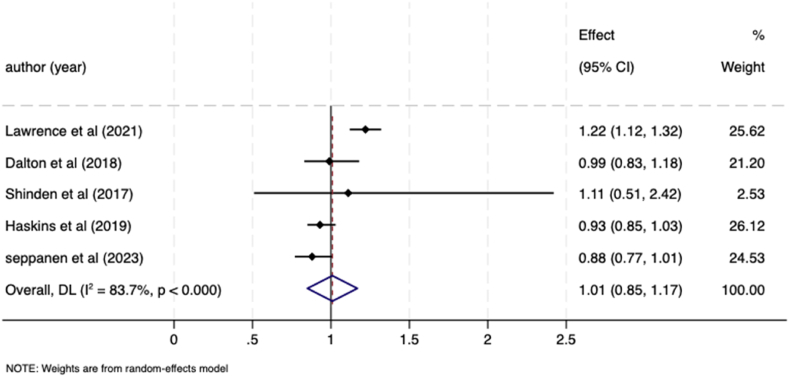
Meta-analysis forest plot showing odds ratio for likelihood of receiving endocrine therapy in breast cancer patients with a pre-existing mental illness compared to patients without a mental illness.

Five studies included data on receipt of endocrine therapy in those with mental illness compared to those with without a mental illness and were eligible for meta-analysis. These studies collectively included data on patients with schizophrenia [30], schizophrenia and depression [28] depression, bipolar disorder, and schizophrenia/psychotic disorders [25,33], and severe mental illness [33]. The pooled OR for receipt of endocrine therapy in those with mental illness compared to those without was 1.01 (95 % CI 0.85–1.17; I^*2*^ = 83.7 %; Tau^2^ = 0.02) (Fig. 6), indicating no significant association between pre-existing mental illness and endocrine therapy.

### 3.9 Surgery

**Fig. 7:**
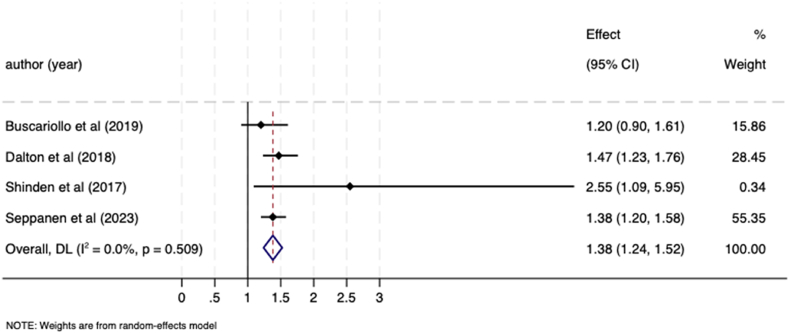
Meta-analysis forest plot showing odds ratio for likelihood of receiving a mastectomy vs breast-conserving surgery in breast cancer patients with a pre-existing mental illness compared to patients without a mental illness.

Five studies examined differences in breast cancer surgery between patients with, and without, a pre-existing mental illness, which included data on patients with depression [26], schizophrenia [32], and psychotic and bipolar affective disorders [33]. Four of these studies were eligible for meta-analysis comparing receipt of mastectomy versus breast-conserving surgery [26,30,32,33]. Women with pre-existing mental illness were significantly more likely to receive a mastectomy; the pooled OR was 1.38 (95 % CI 1.17–1.59; I^*2*^ = 13.8 %; Tau^2=^0.00) (Fig. 7). One study compared rates of implant over autologous reconstruction [21] found that patients with a psychiatric diagnosis of depression, bipolar disorder, or schizophrenia/psychosis were significantly less likely to receive autologous reconstruction compared with implant reconstruction (OR = 0.56, 95 % CI 0.35–0.89).

### 3.10 Treatment delay

One study compared treatment delay between breast cancer patients with and without a pre-existing mental illness [23]. The study found that patients with severe mental illness had an increased risk of treatment delay (defined as ≥60 days) from the original breast cancer diagnosis (Relative Risk (RR) 1.36; 95 % CI 1.06–1.74). Patients with any mental illness experienced an increased risk for adjuvant chemotherapy delay (defined as ≥90 days) from the last operation (RR 1.13; 95 % CI 1.01–1.26).

## 4 Discussion

### 4.1 Summary of main findings

This systematic review and meta-analysis identified 15 eligible studies examining differences in receipt of one or more element of breast cancer treatment in patients with a comorbid mental illness compared to those without. Overall, the findings show there is statistically significant lower odds of utilisation of all elements of breast cancer treatment studied, apart from endocrine therapy, in patients with pre-existing mental illnesses compared to women with no mental illness. Meta-analysis revealed that individuals with a pre-existing mental illness were 21 % less likely than those without a history of mental illness to receive guideline-recommended breast cancer treatment, 44 % less likely to receive chemotherapy and 21 % less likely to receive radiotherapy. In terms of surgery, women with a mental illness were 1.38 times more likely to receive a mastectomy instead of breast-conserving surgery. In addition, a single study comparing implant-based over autologous breast reconstruction found that women with a mental illness were significantly more likely to receive implant-based breast reconstruction [21]. There is also evidence from one study of increased risk of initial treatment delays in patients with a pre-existing mental illness [23].

### 4.2 Explanation of findings and implications for policy and practice

The disparities in receipt of guideline-recommended breast cancer treatment in patients with a mental illness evidenced in this review are not unique to breast cancer; a recent narrative review found that receipt of guideline-recommended treatment was significantly lower across several different cancers, including colorectal, lung, prostate and bladder [35]. Some studies suggest that these treatment disparities may help to explain the higher mortality rate observed in cancer patients with a pre-existing mental illness [32].

The finding from this review that the utilisation of most breast cancer treatments is lower in women with a pre-existing mental illness points towards inequalities or issues with accessibility within the healthcare system. There are several possible explanations for this. In terms of patient-level factors, those with mental illness may experience symptoms such as anxiety, reduced motivation and feelings of hopelessness which may reduce cancer treatment uptake or adherence [36]. In addition, social isolation, which is common in people with mental illness [37], alongside having a low mood, could further contribute to a lack of motivation to undergo certain treatments. Less commonly, in those with more severe mental illness, impairments in cognitive capacity and/or communication skills could result in patients lacking in understanding of cancer treatment regimens, or insight into the importance of them. Patients with symptoms such as delusions, hallucinations and paranoia may even wrongly perceive treatments as a danger to them, which could act as a further barrier to treatment, but again this is likely to be a relative rare reason to explain the reduced rate of treatment in patients with mental illness generally.

When considering provider-level factors, concerns surrounding patient safety and compliance could potentially lead to differential treatment of patients with a pre-existing mental illness. For instance, when a patient receives radiotherapy very precise instructions should be followed – often for several days in a row – to ensure both the treatment effectiveness and safety. In this regard, patients exhibiting distress or a reduced capacity to understand instructions may be less able to manage the treatment and are hence less likely to be offered it. Another example is the use of chemotherapy: in this case, if a patient develops a temperature after chemotherapy, they should present to hospital for further testing. Any delay in this process would be a significant patient safety issue and, it is possible that patients with a mental illness may be perceived as lacking support or awareness that act swiftly on these instructions. Oncologists must therefore carefully weigh up these risks, therefore presenting an additional challenge with potential to influence clinicians' decisions to offer certain guideline-recommended breast cancer treatment.

It is interesting to note that women with a pre-existing mental illness were more likely to receive a mastectomy rather than breast conserving surgery. This may be directly related to screening and diagnosis: individuals with severe mental illness are significantly less likely to take part in breast cancer screening [13], and have been found to delay seeking health care [38]. Consequently, being diagnosed at a more advanced stage (with a larger tumor) may be a contributing factor to the higher rates of mastectomy in this population. Alternatively, previous research has highlighted concerns surrounding treatment compliance in patients with a mental illness [39,40], which may be associated with reduced likelihood of being offered adjuvant chemotherapy or radiotherapy evidenced in this review. The fact that radiotherapy is standard of care following breast-conserving surgery [41] could impact the decision to opt for, or offer, more radical surgery and steer away from more patient-dependent treatment options.

More generally, barriers to optimal care and better integration of oncology and psychiatry services for patients with mental illness should be investigated in qualitative research. This would help to provide a more comprehensive understanding of the underlying mechanisms driving these treatment disparities, and in turn inform potential avenues to which to target future intervention to attempt to reduce them.

### 4.3 Strengths, limitations, and future work

This study goes beyond an earlier systematic review on this topic [42]. As well as being more up-to-date, and including three additional studies, the current review includes meta-analysis for additional outcomes not previously considered, and provides clearer evidence on associations between pre-exisiting mental illness and receipt of adjuvant therapies [42]. We made the decision to combine unadjusted ORs in meta-analyses, due to several critical considerations. Firstly, not all studies eligible for meta-analysis reported adjusted ORs, and combining adjusted with unadjusted effect estimates would not have been appropriate. Secondly, adjusted ORs often incorporate different covariates, so using these for meta-analysis can result in heterogeneity, and inconsistent comparisons across studies. Thirdly, over-adjustment is another concern; the inclusion of irrelevant variables might distort the true effect size. Fourthly, statistical methods for adjustment may also vary, potentially adding further variability unrelated to the actual effect size. By using unadjusted ORs, we sought to provide a more straightforward, transparent, and comparable synthesis of the existing evidence, ensuring that the results are interpretable and replicable without the confounding influence of disparate adjustment models [43]. While the review methods were robust and the findings have important implications for policy and practice, there are several limitations of the evidence-base. Firstly, over half of the included literature was from the USA with most of patients enrolled in national healthcare insurance schemes. Thus, the findings cannot necessarily be generalised to other countries which have publicly-funded healthcare systems, such as the UK. Consequently, this highlights a requirement for further research in a larger range of countries, particularly those with public healthcare systems where research is lacking. There is also particular need for research in lower and middle income countries where there appears to be no research to date. There is also a need to examine the interactions between ethnicity and deprivation with mental illness and their combined impact on care – research has shown a multiplicative impact of these factors [38].

The categorisation and coding of mental illness varied significantly across studies. For example, some studies broadly categorised 'severe mental illness' as a single category, encompassing conditions ranging from severe depression and obsessive-compulsive disorder to bipolar disorder and schizophrenia. It is also worth noting that several otherwise potentially-relevant studies were excluded as they did not distinguish between different classes of mental illnesses, e.g. Ref. [44]. Consequently, subgroup meta-analyses by mental illness type was only feasible for guideline-recommended treatment. Some individual studies which considered different types of mental illness observe that treatment disparities are generally larger for patients with ‘severe mental illnesses’, such as schizophrenia and bipolar disorder, in comparison to those with milder mental illnesses depression and/or anxiety for example [23,45,46]. This review found that the likelihood of receipt of guideline-recommended treatment was very similar for psychotic (OR = 0.77, 95 % CI = 0.61–0.92), and mood disorders (0.78, 95 % CI 0.72–0.84), however these categories do not necessarily correspond with severity.

Of note, no studies explored the association between breast cancer treatment and neurodevelopmental disorders, such as attention deficit hyperactivity disorder (ADHD) and autism spectrum disorder (ASD), and there is a notable absence of research on cancer treatment disparities in such populations. Despite the high prevalence of neurodevelopmental disorders and their common co-existence with mental health difficulties, research on healthcare access disparities, such as those documented for autism [47] should be extended to cancer care. Most studies in this review focus on schizophrenia/psychosis or 'SMI,' with limited data on depression, mood disorders, and anxiety. Future research should include a broader range of mental illnesses; this may provide further clues as to the factors which may lead to these disparities. Given the variability in symptoms across different mental illnesses, the implications for cancer treatment likely differ, highlighting the need to explore barriers and facilitators specific to each mental illness.

Like the mental illnesses included in this review, there have been similar differences in cancer care evidenced for patients with dementia. A recent mixed-studies review concluded that patients with dementia were less likely to receive chemotherapy, radiotherapy, surgery and curative treatment than patients without dementia [48]. This review also found that patients, caregivers and clinicians generally had a preference for less aggressive care and prioritised quality of life over life expectancy. Further research is therefore needed to examine any treatment differences specifically in breast cancer for patients with dementia, and establish treatment guidelines for this unique patient group which account for these patient and caregiver perspectives.

## Conclusion

This systematic review and meta-analysis found that women with pre-existing mental illnesses are significantly less likely to receive guideline-recommended breast cancer treatments, chemotherapy or radiotherapy. Treatment is also more likely to be delayed, and they are more likely to undergo mastectomy instead of breast-conserving surgery. Future research should try to better understand the reasons for these treatment disparities.

Examining the role(s) of systemic issues such as healthcare accessibility, medication interactions, and the segregation of physical and mental health services would be of value.

## CRediT authorship contribution statement

**Katie Elliott:** Writing – review & editing, Writing – original draft, Software, Project administration, Methodology, Formal analysis. **Emily Haworth:** Project administration, Methodology. **Iakov Bolnykh:** Writing – review & editing, Project administration, Methodology. **R. Hamish McAllister-Williams:** Writing – review & editing, Supervision. **Alastair Greystoke:** Supervision. **Adam Todd:** Writing – review & editing, Supervision. **Linda Sharp:** Writing – review & editing, Supervision.

## Declaration of competing interest

The authors declare that they have no conflicts of interest relevant to the content of this systematic review.
