# Supplementary material for: Breast cancer patients with a pre-existing mental illness are less likely to receive guideline-recommended cancer treatment: A systematic review and meta-analysis
Source: Breast. 2024 Dec 7;79:103855. doi: 10.1016/j.breast.2024.103855 (PMC11730251; doi:10.1016/j.breast.2024.103855)
Supplement: Multimedia component 1 [file mmc1.docx]

**Search strategy for OVID**

Breast cancer.mp OR breast neoplasms

AND

Anxiety.mp OR Generalised anxiety disorder.mp OR Generalized anxiety disorder.mp OR Depression.mp OR Depressive disorder*.mp OR Psychotic.mp OR Psychotic disorder*.mp OR Psychosis.mp OR Psychoses.mp OR Schizophrenia.mp OR Schizoaffective disorder*.mp OR Schizophrenic.mp OR Delusional disorder*.mp OR Other psychotic disorder*.mp OR Neurodevelopmental disorder*.mp OR Autistic spectrum disorder*.mp OR ASD.mp OR Autism.mp OR Asperger* Spectrum Disorder.mp OR Asperger*.mp OR Learning disabilit*.mp OR Intellectual disabilit*.mp OR Developmental learning disorder*.mp OR Mental retardation.mp OR Attention-deficit hyperactivity disorder.mp OR ADHD.mp OR Attention Deficit Disorder with Hyperactivity/ OR Attention deficit disorder.mp OR Bipolar disorder.mp

AND

Cancer treatment.mp OR Chemotherapy.mp OR Radiotherapy.mp OR Radiation.mp OR Mastectomy.mp OR Lumpectomy.mp OR Wide local incision.mp OR Endocrine therap*.mp OR Endocrine treatment.mp OR Hormone therap*.mp OR Hormone treatment.mp OR Biological therap*.mp OR Precision therap*.mp OR Trastuzamab.mp OR Anti her 2.mp OR Anti-HER2.mp OR Aromatase inhibitor*.mp OR Tamoxifen.mp

NOT

Survivor.mp OR Survivors.mp OR Survivorship.mp
